# CA-YOLO: An Efficient YOLO-Based Algorithm with Context-Awareness and Attention Mechanism for Clue Cell Detection in Fluorescence Microscopy Images

**DOI:** 10.3390/s25196001

**Published:** 2025-09-29

**Authors:** Can Cui, Xi Chen, Lijun He, Fan Li

**Affiliations:** 1 School of Computer Science and Technology, Xinjiang University, Urumqi 830046, China; cuican1309@163.com; 2 School of Information and Communications Engineering, Xi’an Jiaotong University, Xi’an 710049, China; xi_chen@mail.xjtu.edu.cn (X.C.); lijunhe@mail.xjtu.edu.cn (L.H.)

**Keywords:** clue cells, cell detection, context-awareness, attention mechanism, bacterial vaginosis

## Abstract

Automatic detection of clue cells is crucial for rapid diagnosis of bacterial vaginosis (BV), but existing algorithms suffer from low sensitivity. This is because clue cells are highly similar to normal epithelial cells in terms of macroscopic size and shape. The key difference between clue cells and normal epithelial cells lies in the surface texture and edge morphology. To address this specific problem, we propose an clue cell detection algorithm named CA-YOLO. The contributions of our approach lie in two synergistic and custom-designed feature extraction modules: the context-aware module (CAM) extracts and captures bacterial distribution patterns on the surface of clue cells; and the shuffle global attention mechanism (SGAM) enhances cell edge features and suppresses irrelevant information. In addition, we integrate focal loss into the classification loss to alleviate the severe class imbalance problem inherent in clinical samples. Experimental results show that the proposed CA-YOLO achieves a sensitivity of 0.778, which is 9.2% higher than the baseline model, making the automated BV detection more reliable and feasible.

## 1. Introduction

Bacterial vaginosis (BV) is the most common reproductive tract infection in women, affecting up to 23–29% of the global population [1] and posing a significant health risk to millions [2]. Timely diagnosis and treatment are crucial, as untreated BV can lead to serious complications. These include an increased risk of HPV infection, which is associated with cervical cancer [3,4], and adverse pregnancy outcomes such as miscarriage and premature birth [5]. Therefore, the accurate and efficient detection of clue cells, the core biomarker for BV, is a prerequisite for effective clinical intervention [6].

**Figure 1:**
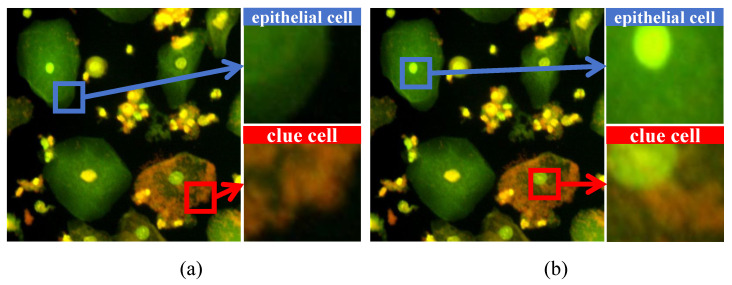
Visual comparison of a clue cell and an epithelial cell in a fluorescence microscopy image. (**a**) Magnified view focusing on the edge difference. (**b**) Magnified view focusing on the surface texture difference. The red boxes indicate the clue cell, while the blue boxes indicate the epithelial cell.

However, automated clue cell detection faces several challenges due to several intrinsic factors. As illustrated in Figure 1, clue cells are Gardnerella-infected epithelial cells. They are thus highly similar to their normal counterparts in macroscopic size and shape. The truly discriminative information lies in subtle microscopic features [7,8]. For instance, the edges of clue cells exhibit a fuzzy, irregular granularity, whereas normal epithelial cells have smooth, clear boundaries (Figure 1a). This edge degradation often presents as high-frequency noise and is easily lost during standard convolution operations. Furthermore, the surface of clue cells is covered with a dense layer of Gardnerella, creating complex texture information. In contrast, normal epithelial cells have a uniform and smooth surface (Figure 1b). Capturing this bacterial distribution texture is fundamental for accurate classification. A final challenge is the severe class imbalance in clinical samples, where normal epithelial cells vastly outnumber clue cells. This can cause detection models to overlook the minority clue cell class. Consequently, an effective algorithm must excel at capturing fine-grained edge and texture details while robustly handling data imbalances.

While deep learning has advanced medical image analysis [9,10,11], existing object detection methods remain ill-suited for the specific demands of clue cell detection. Two-stage detectors like Faster R-CNN, though accurate in localization, are too computationally intensive for real-time clinical use and were not designed to capture microscopic features [12,13,14]. Mainstream one-stage detectors, such as the YOLO series, are faster, but their standard backbones tend to lose critical high-frequency information during downsampling, resulting in low sensitivity [15]. Even specialized cell detection models like CAF-YOLO [16] and YOLO-TCT [17] fall short. This is because they are optimized for different pathological features, such as cell nuclei characteristics or nucleus–cytoplasm ratios [18], and are not tailored to the unique edge and texture signatures of clue cells. As a result, there is an urgent need for an end-to-end model designed specifically for this task.

To overcome these limitations, we propose CA-YOLO, a deep learning model specifically engineered for high-sensitivity clue cell detection. Our model employs a synergistic strategy of targeted feature extraction and loss optimization. The main contributions are as follows: A novel context-aware module (CAM) is designed to capture the complex surface texture of clue cells. By modeling long-range dependencies across the feature map, CAM integrates local bacterial patterns into a global, semantic representation, effectively distinguishing them from the smooth surfaces of normal cells. A shuffle global attention mechanism (SGAM) is proposed to address the challenge of fuzzy cell edges. By combining spatial and channel attention with a large receptive field, SGAM accurately locates and enhances weak edge features while suppressing background noise. The integration of focal loss directly tackles the class imbalance problem, working in concert with our feature enhancement modules to create a complete end-to-end solution. Experimental results show that our CA-YOLO model achieves a sensitivity of 0.778, significantly improving upon baseline models and effectively reducing the risk of missed diagnoses in a clinical setting.

## 2. Related Work

### 2.1. Cell Detection

Due to its efficiency and real-time capabilities, the YOLO framework has been extensively applied to cell detection tasks, covering multiple cell types such as blood cells [19], cancer cells, and clue cells [20]. For instance, Wu et al. proposed the EB-YOLO model, which incorporated ShuffleNet as a lightweight backbone alongside the Convolutional Block Attention Module (CBAM) and the Adaptive Spatial Feature Fusion (ASFF) module. This architecture maintained high detection accuracy while reducing computational costs [21]. Similarly, Haq et al. integrated YOLOv2 with ResNet-18 and ResNet-50 for the detection of colorectal cancer cells, demonstrating particular effectiveness in identifying overlapping cells [22].

In the domain of clue cell detection, several approaches have also been introduced. Guo et al. developed a classification method based on multi-scale texture energy descriptors to extract cellular features, employing an SVM classifier to differentiate between cell types [23]. Yu et al. presented a Convexified Coupled Active Contour (CCAC) model that exploited structural relationships in immunofluorescence images to jointly segment clue cells, nuclei, and bacteria [24]. More recently, Chen et al. introduced the Multi-Scale Perceptual YOLO (MSP-YOLO), which integrated a super-resolution branch and an attention-based feature fusion module. This was designed to enhance the network’s sensitivity to the subtle features of clue cells and trichomonas [25]. Despite these advances, existing methods for clue cell detection still face limitations in sensitivity, primarily due to indistinct morphological features and significant class imbalance.

### 2.2. Context Information Enhancement

Traditional object detectors primarily rely on local features for object localization. However, in scenes with complex backgrounds or blurry targets, this reliance on local features can result in false detections or missed targets [26]. Incorporating a context information enhancement module enables a model to capture the global image context. This provides a more comprehensive understanding of the scene and improves detection accuracy and robustness [27].

Context-based modules have achieved promising results in object detection. For example, CGNet integrates multi-scale features through context-guided blocks, improving the recognition of slender structures [28]. Non-local networks efficiently model global information by capturing long-range dependencies, which particularly benefits small-object detection [29]. Additionally, the fusion of multi-scale features with global context has become a key research focus. Cui et al. proposed the Multi-scale Global Feature Aggregation Network (MGA-Net), which aggregates global features across different scales to improve adaptability to small objects and complex backgrounds [30]. In summary, context-enhancement modules are essential for optimizing detection performance in complex scenarios.

### 2.3. Attention Mechanisms

The attention mechanism dynamically assigns weights to emphasize key features, enhancing performance while reducing computational complexity in image processing [31]. It has been widely applied in various visual tasks, including image classification [32], object detection [33], semantic segmentation [34], 3D vision [35], etc. Common attention mechanisms are categorized into three types: channel attention [36,37], spatial attention [38,39], and a combination of both [40,41]. Channel attention determines which features to emphasize by adjusting the weights of each channel. Mo et al. proposed a U-Net-based network that incorporated the Efficient Channel Attention (ECA) mechanism to address blurred-edge segmentation [42]. Separately, Lu et al. introduced an enhanced channel attention residual block in their ECARN model to further improve performance [43]. Spatial attention, on the other hand, dynamically adjusts the weights of different spatial positions. This allows the model to emphasize key areas and suppress background noise. Verma et al. proposed a spatial attention mechanism to dynamically highlight key anatomical structures in fetal ultrasound images [44]. Liu et al. introduced the MCSA-HRNet, which employed dedicated attention blocks to improve focus on target-related regions [45]. However, both mechanisms have limitations. Channel attention can be less sensitive to fine image details, while standard spatial attention may struggle to capture the fine-grained features necessary for recognizing the indistinct edge morphology of clue cells.

## 3. Materials

The fluorescence microscopy image dataset used in this study was collected by Suzhou Tumo Medical Technology Co., Ltd. (Suzhou, China). It comprised 2314 images from 480 patients, with each patient contributing 2 to 8 images. For each patient, gynecological vaginal discharge samples were collected, and slides were prepared through a standardized process of dilution and staining. The prepared slides were then imaged using a fully automated immunofluorescence detection device (model YG02). The original image resolution was 2736 × 1824 pixels. Due to computational constraints and the need for efficient slide analysis, a standard preprocessing pipeline was initially applied, where original images were down-sampled to 1368 × 912 pixels and further cropped into four 684 × 456-pixel images. However, for the specific purposes of this research, we adopted a different strategy. Each original image was randomly cropped to 1024 × 1024 pixels and then down-sampled to a final input size of 512 × 512 pixels. Ultimately, our final dataset consisted of 2314 images. Among these, 455 images contained clue cells, while 1859 did not. The dataset was partitioned into a validation set of 576 images (containing 231 clue cell instances) and a test set of 477 images (containing 144 clue cell instances). The primary goal of this study was to detect these clue cell targets. As visually detailed in Figure 1, the main challenge lay in distinguishing clue cells from normal epithelial cells based on subtle differences in their edge characteristics (Figure 1a) and surface texture (Figure 1b).

## 4. Theory

### 4.1. The YOLOv8 Architecture

The YOLOv8 algorithm comprises three main components: the backbone, neck, and detection head [46]. The backbone network is responsible for initial feature extraction from the input image. It primarily utilizes CBS and C2f modules, progressively down-sampling the image to generate a hierarchy of feature maps at different spatial resolutions. The neck component, implemented as a Path Aggregation Network (PANet), is designed to fuse feature maps from different levels of the backbone. By aggregating features from both top-down and bottom-up pathways, the neck produces feature representations that are rich in both semantic and spatial information, enhancing the model’s ability to detect objects at various scales. The final predictions are generated by the three detection heads with different scales. YOLOv8 employs a decoupled head structure, where classification and regression tasks are handled by separate branches. This design allows each task to be optimized independently, improving overall detection accuracy.

### 4.2. Standard Loss Function of YOLOv8

The overall loss function of the YOLOv8 model is a sum of two components: classification loss $L_{cls}$ and bounding box regression loss $L_{bbox}$. It can be expressed as

(1) $$L_{YOLOv8}=L_{cls}+L_{bbox}.$$

The classification loss is typically calculated using binary cross-entropy (BCE) loss for each potential object. The BCE loss for a single prediction is given by

(2) $$L_{BCE}=-y\log\hat{y}-\left(1-y\right)\log\left(1-\hat{y}\right),$$

where y∈{0,1} is the ground-truth label, with 1 indicating a positive sample and 0 indicating a negative sample. $\hat{y}$ is the model’s predicted probability.

The bounding box regression loss is a combination of the Complete Intersection over Union (CIoU) loss and the Distribution Focal Loss (DFL). This composite loss aims to accurately regress the coordinates of the bounding box. It is formulated as

(3) $$L_{bbox}=L_{CIoU}+L_{dfl}.$$

The CIoU loss accounts for the overlap area, central point distance, and aspect ratio consistency between the predicted and ground-truth boxes. DFL treats the continuous box coordinate regression as a discrete classification problem over a range of values, which helps in learning a more accurate and flexible bounding box representation.

## 5. Method

**Figure 2:**
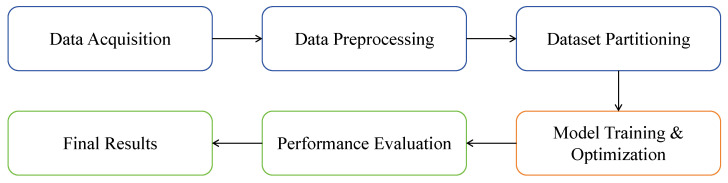
The overall workflow of the proposed CA-YOLO model.

**Figure 3:**
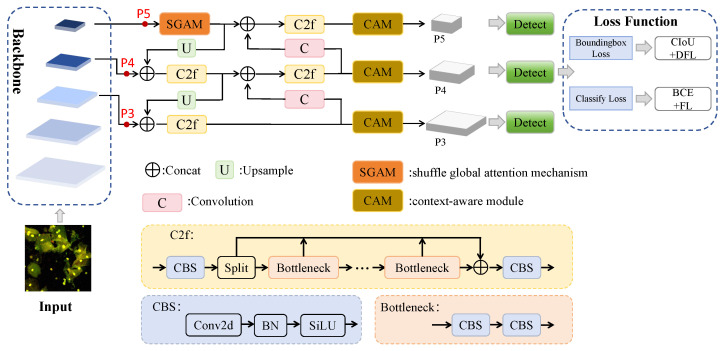
Overview of the proposed CA-YOLO framework.

The overall workflow of our research is illustrated in Figure 2. The process begins with data acquisition and preprocessing, followed by patient-level partition of the dataset. The core of our approach is the training and optimization of our proposed CA-YOLO model, followed by a rigorous performance evaluation to generate the final detection results. This section details the key components of our model. The CA-YOLO model, as shown in Figure 3, is built on the YOLOv8 framework and takes a 512 × 512 pixel image as input. Our key innovation lies in the introduction of two collaborative modules within the neck structure to detect clue cells: the CAM is designed to capture the complex distribution patterns of bacteria attached to the cell surface. The SGAM enhances information interactions across channels and spatial locations, significantly improving the model’s ability to detect fuzzy cell boundaries. After being enhanced by these modules, the features are passed to the detection head for final prediction. The model’s loss function combines CIoU and distributional focal loss for bounding box regression, and binary cross-entropy and focal loss for classification. The following subsections describe each innovative component in detail.

### 5.1. Context-Aware Module

Distinguishing between clue cells and epithelial cells is challenging due to their morphological similarities and variations in bacterial distribution on their surfaces. The context-aware module (CAM) captures global contextual information from image features, enhancing the model’s ability to differentiate between these cell types by analyzing the spatial distribution of attached bacteria. Additionally, CAM is lightweight and can be integrated into multiple network layers, significantly improving detection accuracy while imposing minimal computational overhead.

**Figure 4:**
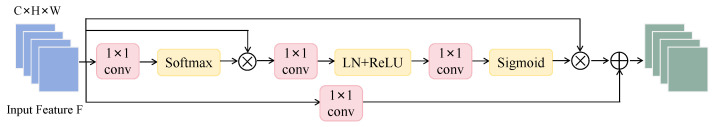
The details of the context-aware module.

CAM processes image feature maps through a series of structured operations. A 1 × 1 convolution adjusts the number of channels without altering spatial dimensions, followed by Softmax normalization to convert outputs into probability distributions. The Softmax output is used to generate a weighted feature representation by performing element-wise multiplication with the input feature map. A second convolution refines the channel dimensions, followed by layer normalization (LN) to stabilize training. A third convolution processes the feature map, after which the output is transformed into probability values between 0 and 1 using the Sigmoid activation function. The probability values are applied element-wise to the weighted input feature map, enhancing important channel features. Finally, the weighted results are added to the original input feature map, generating an output feature map rich in global contextual information (Figure 4).

### 5.2. Shuffle Global Attention Mechanism

**Figure 5:**
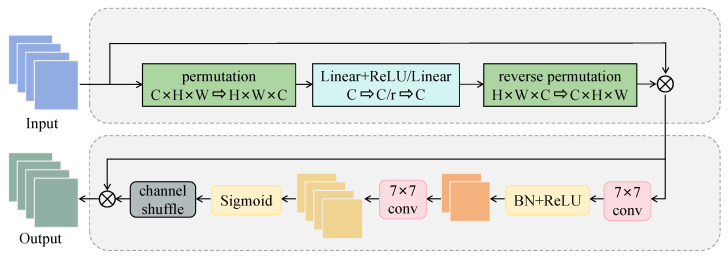
The details of shuffle global attention mechanism.

We propose the shuffle global attention mechanism (SGAM) to guide the network in focusing on cell contour features. The module structure is illustrated in Figure 5. SGAM enhances the model’s ability to focus on key features by integrating channel attention and spatial attention, and incorporates channel shuffle at the end to promote more effective channel mixing and improve the network’s expressive capacity.

The input feature map of SGAM is denoted as $X\in R^{C\times H\times W}$, where C, H, and W represent the number of channels, height, and width, respectively. The channel attention first transposes the input tensor from C × H × W to H × W × C to facilitate the calculation of attention weights. Then, two fully connected layers reduce the number of channels and restore to C, allowing the model to learn channel importance. Next, the channel attention weights are rearranged into a C × H × W shape and multiplied element-wise with the original input. The channel attention weights are computed as

(4) $$A_{c}=\sigma \left(W_{2}\cdot \delta \left(W_{1}\cdot P\left(X\right)\right)\right),$$

where P(·) represents permutation operation, $W_{1}$ and $W_{2}$ represent fully connected layers, and δ(·) and σ(·) denote the ReLU and Sigmoid activation functions, respectively. The output of the channel attention is obtained as $X_{CA}\;=\;X\;⊙\;P(A_{c}$), where ⊙ denotes element-wise multiplication.

Given that clue cells are relatively large targets in images, the use of 7 × 7 convolution instead of smaller kernels in spatial attention can have a wider receptive field and better capture regional cell contour features, which is crucial for distinguishing clue cells and surrounding epithelial cells. The second 7 × 7 convolution further refines the extracted features, enhances spatial information representation, and generates the spatial attention map. In addition, we have designed channel shuffle for spatial attention maps that can promote cross-channel information exchange, effectively utilize the dependency relationships between channels, avoid information redundancy or loss, and make it easier to focus attention on areas with strong pathological features, thereby effectively improving sensitivity. The spatial attention weights are computed as

(5) $$A_{s}=\sigma \left(f_{2}\left(\mathrm{BN}\left(\delta \left(f_{1}\left(X_{CA}\right)\right)\right)\right)\right),$$

where $f_{1}\left(\cdot \right)$ and $f_{2}\left(\cdot \right)$ denote 7 × 7 convolutions, δ(·) is the ReLU function, BN is batch normalization, and σ(·) is the Sigmoid function. The final output features after spatial enhancement are computed by $X_{SA}\;=\;X_{CA}\;⊙\;S\left(A_{s}\right)$, where S(·) denotes channel shuffle.

### 5.3. Loss Function

While the standard YOLOv8 loss function is effective for general object detection, it is not optimized for the severe class imbalance inherent in clinical clue cell samples, where normal epithelial cells vastly outnumber clue cells. To address this, we enhance the classification loss component of the total loss function.

The total loss of our CA-YOLO model is defined as

(6) $$L_{total}\;=\;L_{cls\_enhanced}\;+\;L_{bbox},$$

where the regression loss $L_{bbox}$ remains the same as in the standard YOLOv8 (Equation (3)), but the classification loss is specifically enhanced.

Our key modification is the integration of focal loss into the classification loss, resulting in an enhanced loss term $L_{cls\_enhanced}$:

(7) $$L_{cls\_enhanced}\;=\;L_{BCE}\;+\;L_{FL},$$

where $L_{BCE}$ is binary cross-entropy loss (Equation (2)). Focal loss adjusts the weights of minority samples that are difficult to classify, forcing the model to pay more attention to clue cells. Focal loss is defined as

(8) $$L_{FL}\left(p_{t}\right)\;=\;-\alpha_{t}{\left(1\;-\;p_{t}\right)}^{\gamma }\log p_{t},$$

where $p_{t}$ is the model’s estimated probability for the ground-truth class. The focusing parameter γ down-weights the loss assigned to well-classified examples (e.g., easy-to-identify epithelial cells), while the balancing parameter $\alpha_{t}$ directly addresses class imbalance by assigning a higher weight to the minority class. By incorporating focal loss, our model becomes significantly more sensitive to the challenging clue cells, which is critical for achieving reliable automated BV detection.

## 6. Experiments

### 6.1. Setup

This subsection outlines the experimental setup for evaluating the performance of the proposed method, including the dataset partitioning strategy, preprocessing steps, and training and testing configurations, as well as the experimental design and evaluation metrics.

#### 6.1.1. Implementation Details

In order to conduct a robust and unbiased evaluation of the model, we adopted a two-step data splitting strategy. First, the 2405 images were randomly divided at the patient level into a training set (80%) and a test set (20%), ensuring that the test set remained isolated throughout the development process. The training set was then further divided into a training set (70%) and a validation set (30%). In the end, 1352 images were used for training, 576 for validation, and 477 for testing. All subsequent experiments used the same training and test sets.

To address the limited training data and class imbalance, we employed data augmentation strategy during training. This included geometric transformations (random rotation, scaling, flipping, translation, and mild shearing) and compound augmentations (mosaic, MixUp, and Copy-paste). Data augmentation was applied exclusively to the training set, leaving the validation and test sets unaltered, which only increases the training cost but not the inference cost [47].

In the training stage, we applied random search to optimize three key hyper-parameters: learning rate, batch size, and weight decay [48]. The hyper-parameter search space we defined is shown in Table 1, and we conducted 20 independent training experiments by randomly sampling combinations from this space. The model’s F1 score on the validation set was used as the primary evaluation metric. The experimental results indicate that the best performance was achieved with a learning rate of 0.001, a batch size of 64, and a weight decay of 0.0005. This configuration was used as the default setting in all subsequent experiments. The model was trained using the Adam optimizer for 500 epochs. To mitigate overfitting, we implemented an early stopping strategy.

To ensure the stability and reliability of findings, all experiments on the hold-out test set were repeated five times with different random seeds, and the mean and standard deviation were calculated as the final performance metrics. Furthermore, to validate that the observed improvements are statistically significant, we conducted statistical analysis, including Bootstrap resampling and McNemar’s test, which is presented in Section 6.5.2.

All experiments were conducted on a machine equipped with a Tesla V100 GPU. The training framework used YOLOv8-s and was implemented using PyTorch 1.11.0 and Python 3.11.5.

#### 6.1.2. Experimental Design

We first conducted a model analysis, focusing on several key factors: the impact of the hyper-parameter γ in focal loss, the optimal insertion position of the SGAM, and the comparison of the effects of SGAM with other attention mechanisms (such as Squeeze-and-Excitation block (SE) [36], Convolutional Block Attention Module (CBAM) [40], Efficient Channel Attention module (ECA) [49], and Simple Attention Module (SimAM) [50]). Then, we analyzed the relationship between the loss function, epochs and metrics during training. To further verify the performance of the model, we conducted ablation studies to evaluate the contribution of each component of CAM, SGAM, and focal loss.

Subsequently, we conducted comparison studies of CA-YOLO with various object detection methods. The comparison models include YOLOv11-s [51]; CA-YOLO (v11), which replaces the baseline of CA-YOLO with YOLOv11-s; and cell detection models such as MSP-YOLO [25], CAF-YOLO [16], and YOLO-TCT [17]. In order to demonstrate the performance of the model, we selected representative samples to visualize the detection results, plotted the ROC curves and PR curves of the model, and performed statistical validation with the baseline to prove the effectiveness of CA-YOLO.

Finally, we tested the generalization ability of the model on the public blood cell count and detection dataset (BCCD) [52]. The BCCD dataset contains 364 microscope images of 640 × 480 pixels, covering cell types such as red blood cells (RBCs), white blood cells (WBCs), and platelets, with a total of 4888 labels. The dataset is divided into a training set (255 images), a validation set (73 images), and a test set (36 images). On this dataset, we use object-level evaluation metrics such as precision, recall, and F1 score for evaluation.

#### 6.1.3. Evaluation Metrics

We selected four metrics to evaluate clue cell detection in fluorescence microscopy images: Sensitivity (SEN), Specificity (SPE), Accuracy (ACC), and F1 score (F1). SEN reflects the model’s ability to correctly identify positive instances, with a higher SEN indicating a reduced rate of missed detections. SPE measures the proportion of true negatives accurately identified, where a higher SPE corresponds to fewer false positives. ACC assesses the overall classification performance of the model across both positive and negative classes. The F1 score is the harmonic mean of precision and recall, taking into account both missed detections and false detections, and is particularly useful for dealing with class imbalance. SEN, SPE, and ACC assess detection performance at the image level, while the F1 score evaluates performance at the object level. We diagnosed a true positive (TP) when the clue cells occupied more than 20% of the area of both epithelial cells and clue cells in a single image. The formulas for SEN, SPE, ACC, and F1 score are defined as follows:

(9) $$SEN=\frac{TP}{TP+FN},$$

(10) $$SPE=\frac{TN}{TN+FP},$$

(11) $$ACC=\frac{TP+TN}{TP+TN+FP+FN},$$

(12) $$F1\;score=2\times \frac{Precision\times Recall}{Precision+Recall},$$

(13) $$Precision=\frac{TP}{TP+FP},$$

(14) $$Recall=\frac{TP}{TP+FN},$$

where TP, TN, FP, and FN denote true positives, true negatives, false positives, and false negatives, respectively.

### 6.2. Model Analysis

#### 6.2.1. Hyper-Parameter γ

In the CA-YOLO model, the focal loss function plays a key role in addressing the class imbalance problem in the clue cell detection. The performance of focal loss is mainly modulated by two hyper-parameters: the balancing factor α and the focusing parameter γ. We introduce the setting strategy of α and analyze the impact of γ on the model performance.

**Figure 6:**
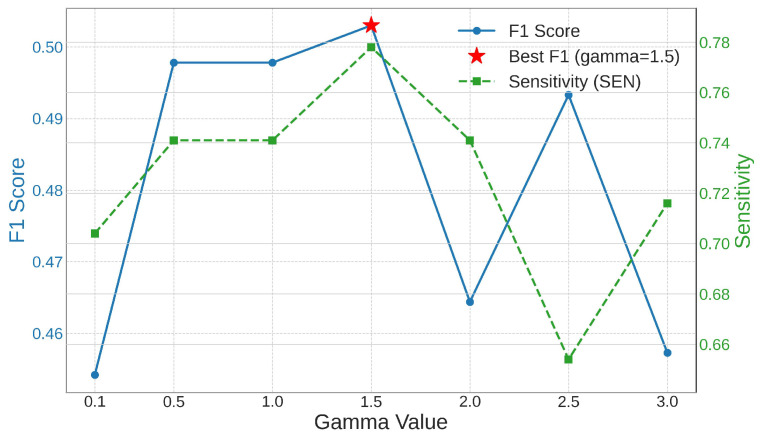
Impact of γ on F1 score and SEN.

The α is used to statically balance the importance of different classes. We determine the weight of each class based on the inverse frequency of the corresponding class in the training set. This data-driven approach assigns higher weights to rarer clue cells, allowing the model to pay more attention to them during training. To ensure fair and consistent experimental evaluation, the pre-computed α remains unchanged in all subsequent experiments. The γ enables the model to focus on misclassified samples by dynamically adjusting the loss contribution of easy and difficult samples. To explore its optimal value, we conducted a sensitivity analysis. We trained the full CA-YOLO model with different γ values ranging from 0.1 to 3.0, keeping all other settings and α unchanged. Figure 6 shows the changes in SEN and F1 score on clue cells under different γ values of the model. As shown in Figure 6, as the γ increases from 0.1 to 1.5, the SEN and F1 score increase steadily and reach a peak at γ = 1.5. This shows that a moderate focusing effect is beneficial to our task, which can effectively reduce the loss from easy-to-classify background samples and help the model prioritize more difficult samples, thereby improving its precision without significantly affecting the recall. However, when the γ increases further, the model performance begins to decline. This is because a large γ overly suppresses the loss of classified samples, which may hinder the overall learning process and stability of the model.

Based on this comprehensive analysis, we choose γ = 1.5 as the optimal setting for the CA-YOLO model to ensure the best performance in comparative studies and ablation experiments.

#### 6.2.2. Insertion Position of the SGAM

We conducted experiments to determine where to insert SGAM in the backbone network to obtain the best detection performance. “3_layers_P345” means applying the SGAM after P3, P4, and P5 in Figure 3; “1_layer_P5” means applying the SGAM solely after P5. The experiments in Table 2 show that placing SGAM only on the deepest feature map (P5) is better than applying it to all levels (P3, P4, P5). This is because global structural information such as cell contours should be captured from high-level semantic features, which have larger receptive fields. Applying SGAM to shallow high-resolution feature maps may introduce noise, forcing the model to search in irrelevant long-range dependencies, thereby degrading performance.

#### 6.2.3. Different Attention Mechanisms

To validate the effectiveness of the proposed SGAM, we conducted comparative experiments between SGAM and four representative attention mechanisms: the SE [36] is the earliest and most widely adopted channel attention structure; the CBAM [40] models both channel and spatial attention sequentially and is commonly used in medical image tasks; the ECA [49] is a lightweight attention design that eliminates fully connected layers and reduces parameters; and the SimAM [50] is a parameter-free spatial attention approach inspired by neuroscience energy functions, known for its simplicity and efficiency. The results in Table 3 verify the superiority of SGAM. SGAM can more effectively simulate long-range dependencies through its permutation and enhanced global receptive field and accurately capture the subtle differences in cell edge morphology, while the channel shuffle component enhances information flow and avoids information bottlenecks.

### 6.3. Training Process Analysis

**Figure 7:**
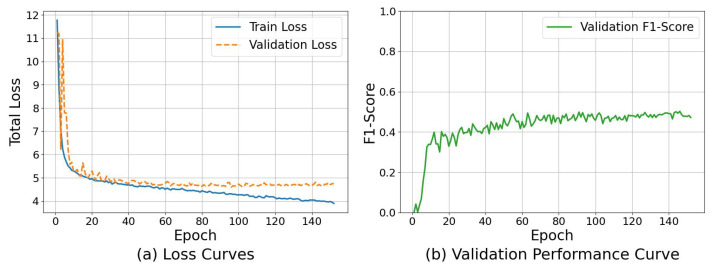
CA-YOLO training process analysis.

We explain the relationship between loss functions, evaluation metrics, and training epochs to demonstrate the stability and effectiveness of CA-YOLO. Figure 7 shows a visualization of the training process. The performance of CA-YOLO is evaluated at the image and object levels, and the image-level metrics SEN, SPE, and ACC are calculated once on the test set through an independent evaluation script after the final model is determined. Therefore, during the training process, we focus on the object-level evaluation metric the F1 score.

As shown in Figure 7a, both the training loss and the validation loss drop rapidly in the initial stage of training, indicating that the model effectively learns the features of the clue cells from the training data. After the stage, while the training loss continues to gradually decrease, the validation loss decreases slowly and gradually flattens out. This difference between the training and validation loss curves indicates that the model has reached its best generalization point and begins to show signs of slight overfitting on the training data. The validation performance curve in Figure 7b further confirms this observation. In the initial stage of training, the F1 score grows rapidly, corresponding to the initial sharp drop in loss, and gradually stabilizes after about 40–50 epochs. By analyzing the training process, we select the model corresponding to the highest F1 score on the validation set as the optimal model for clue cell detection, which ensures that the model used for the final evaluation has the best generalization ability on the test set.

### 6.4. Ablation Study

To verify the effectiveness of each module in the proposed CA-YOLO network, the following comparison experiments were conducted: removing the SGAM, the CAM, and the focal loss, respectively. The results are shown in Table 4. After removing SGAM, SEN decreased by 1.2%, which shows that the SGAM is effective in improving the detection ability of the model. After removing CAM, SEN decreased by 1.7% and F1 score decreased by 1.5%. This not only proves the effectiveness of CAM, but the extent of the SEN decrease suggests that CAM contributes most to the improvement of model performance in clue cell detection. After removing focal loss, SEN decreased by 1.3% and F1 score decreased by 2.3%. This proves that increasing the attention to a few samples can improve the sensitivity of clue cell detection. The ablation results prove that the SGAM, CAM, and focal loss strategies are indispensable. Each component contributes to the final performance, among which CAM is particularly prominent in improving sensitivity. The optimal detection performance is achieved under the synergy of these components.

### 6.5. Comparison Study

#### 6.5.1. Comparison with Other Detection Methods

**Figure 8:**
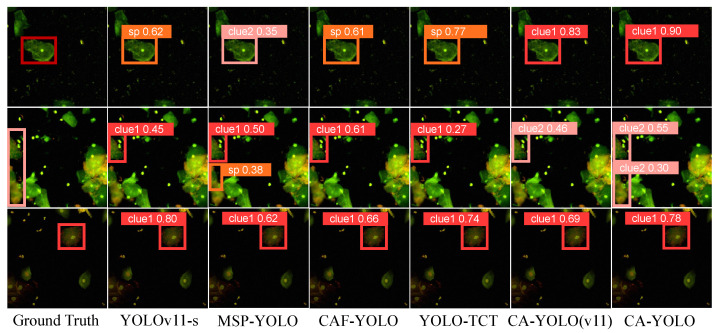
The visualization results of different detection models.

In Table 5, we compare the performance of CA-YOLO with various object detection methods in clue cell detection. The comparison models include YOLOv11-s [51]; CA-YOLO (v11), with YOLOv11-s replacing the CA-YOLO baseline; and cell detection models such as MSP-YOLO [25], CAF-YOLO [16], and YOLO-TCT [17]. All models are trained and tested on our clue cell dataset under the same conditions. MSP-YOLO guides the network to learn small feature differences through super-resolution reconstruction, which improves the performance of small object detection. CAF-YOLO introduces the channel attention and spatial attention fusion module to effectively enhance the accuracy of blood cell detection. YOLO-TCT solves the class imbalance problem through an improved loss function, improving the performance of long-tail cervical cell detection. From the results, it can be seen that after replacing the CA-YOLO baseline with YOLOv11-s, the algorithms effect is not much different, which shows that the effectiveness of the module designed for clue cell features is not limited to the specific architecture of YOLOv8-s, but shows good generalization ability and consistent performance improvement on different backbone networks. Compared with other cell detection algorithms, CA-YOLO performs best in terms of SEN, reaching 0.778, while maintaining high SPE and ACC. This proves that the model is effective in identifying clue cells and reducing missed detections and is also superior in comprehensive performance. It is a detection model that is more suitable for this task. The visualization results of different detection models are shown on Figure 8.

**Figure 9:**
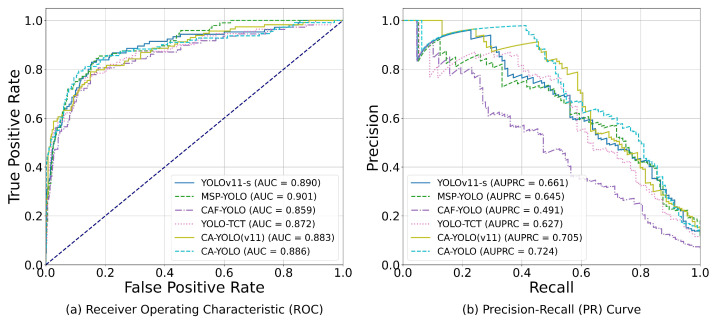
Performance comparison of different models on ROC and PR curves.

In order to conduct a more comprehensive and threshold-independent evaluation of the CA-YOLO model, we plotted the Receiver Operating Characteristic (ROC) curves and Precision–Recall (PR) curves on the test set, as shown in Figure 9. As can be seen from the ROC curve, the Area Under Curve (AUC) values of all models are high and very close together, indicating that these detection models can effectively distinguish clue cells from the background. However, since the ROC curve is insensitive to a large number of true negative samples (i.e., correctly identified background areas) in the dataset, its discrimination in class imbalance scenarios is relatively limited. Considering that our task is to detect clue cells, a sparse target that accounts for a small proportion of the image and is easily confused with the background or other cells, the PR curve, as the gold standard for measuring the performance of the model in identifying positive samples, is more advantageous in this scenario. As can be seen from the PR curve, the CA-YOLO model achieved the highest AUPRC value of 0.724. More importantly, the PR curve of the CA-YOLO model is above other curves in most recall intervals, which shows that CA-YOLO can complete the task with a lower false detection rate, whether under a conservative detection strategy (low recall) that requires high precision or a comprehensive search strategy (high recall) that pursues a high detection rate. This feature is crucial for clinical auxiliary diagnosis application scenarios that require high reliability, because it directly affects whether accurate and reliable reference information can be provided to doctors.

In summary, CA-YOLO performs well in clue cell detection, especially in terms of PR curve performance, indicating that it can better handle the challenges of class imbalance and improve recall while ensuring high precision. This proves the reliability of CA-YOLO in the clinic.

#### 6.5.2. Statistical Validation

To verify that the performance advantage of the CA-YOLO model over the baseline model YOLOv8-s is not due to random factors, we introduced two statistical test methods for analysis. First, we applied the Bootstrap resampling test to estimate the 95% confidence interval (CI) of the difference in accuracy between the two models. This method can quantify the credibility of the observed performance improvement. The CA-YOLO model has an accuracy of 90.20%, while the baseline model has an accuracy of 85.61%, with an average difference of +4.59%. Second, we used McNemar’s test. This test determines whether there is a significant difference in the error rates of the two models by analyzing samples where the prediction results of the two models are inconsistent (i.e., one model is correct and the other is wrong). In 477 test images, the CA-YOLO model corrected 34 errors of the baseline model and only introduced 13 new errors. This significant asymmetry indicates that the CA-YOLO model performs better. The key results of the two tests are summarized in Table 6.

As shown in Table 6, both statistical test methods provide strong evidence. The results of the Bootstrap test show that the 95% confidence interval of the CA-YOLO model’s accuracy improvement is [+1.75%, +7.43%]. Since this interval is completely above zero, it can be concluded that the performance advantage of the CA-YOLO model is statistically significant. The *p*-value obtained by McNemar’s test is 0.0035 (i.e., p < 0.01), which enables us to reject the null hypothesis that “the error rates of the two models are the same”, further confirming that the performance of the CA-YOLO model is significantly better than that of the baseline model.

In summary, these statistical analysis results jointly prove that the superiority of the CA-YOLO model in the clinical clue cell detection task is real and robust, rather than an accidental experimental result.

### 6.6. Cross-Dataset Generalization Validation

In order to verify the generalization ability and robustness of the proposed CA-YOLO model under different tasks and data distributions, we selected the commonly used public BCCD dataset [52] for experiments and compared it with the mainstream detection model YOLOv8-s and the blood cell detection model BC-YOLO [53].

The detection results are shown in Table 7. The CA-YOLO model has a precision of 0.553, a recall of 0.926, and an F1 score of 0.693 on the BCCD dataset, all of which are higher than YOLOv8-s, indicating that its overall detection performance is better. In contrast, as a model optimized specifically for blood cell detection, BC-YOLO performs best in all three metrics. However, CA-YOLO can achieve performance close to that of a dedicated model in cross-dataset testing, which proves that our proposed model can effectively extract common cell features rather than just fitting the patterns of a specific dataset, fully demonstrating its good generalization ability and robustness.

In summary, the CA-YOLO model is not only competitive in the clue cell detection task, but also can achieve better results than mainstream models in blood cell detection tasks with different data distributions, thus verifying its practical value in cell detection tasks.

## 7. Discussions

We compare the proposed CA-YOLO model with the baseline model YOLOv11-s [51]. Although YOLOv11-s performs well in general tasks, it is less effective for the specific detection of clue cells. MSP-YOLO [25] focuses on super-resolution and is more suitable for detecting small targets; CAF-YOLO [16] adopts a CNN–Transformer hybrid method but is mainly used for blood cell detection; YOLO-TCT [17] combines Hard Polarized Self Attention (HPSA) and an improved loss function to perform better in long-tail neck cell detection. CA-YOLO performs particularly well in clue cell detection by simultaneously modeling cell surface contextual features and edge morphological features. CA-YOLO has the highest SEN but slightly lower SPE and ACC. In contrast, YOLOv11-s adopts a conservative strategy, leading to a higher SPE but a lower SEN. This trade-off is not a defect of the model, but meets the needs of clinical practice. In medical diagnosis, the risk of missed diagnosis is usually greater than that of misdiagnosis. Therefore, CA-YOLO could be a more effective auxiliary diagnostic tool in clinical screening while ensuring high sensitivity. Figure 8 shows the detection results of these models. As shown in Figure 8, in confusing scenes (row 1), most of the comparison models made mistakes for clue cells that are easily misjudged as epithelial cells, while CA-YOLO can identify them with a high confidence of 0.90. In scenes with dense cells and unclear features (rows 2–3), other models generally encounter problems involving missed detection, false detection, or low confidence, while CA-YOLO can provide correct and high-confidence detection results.

A key finding of our study is that while SEN improved significantly, the F1 score was only 0.503. This is due to a trade-off between precision and recall. This trade-off is not a flaw in the model, but rather a response to the clinical need to prioritize reducing missed diagnoses. In diagnostic screening, missed diagnoses are far more severe than false positives, leading to higher false positive rates (lower precision) because some epithelial cells with ambiguous features may be misclassified as clue cells. The high sensitivity of the CA-YOLO model allows the majority of potential clue cells to be flagged for clinician review. While this may include some false positives, this aligns with the primary goal of this screening tool: minimizing the risk of missing potential bacterial vaginosis cases.

The practical application of CA-YOLO lies in its integration into clinical diagnostic workflows. Regarding efficiency, our model achieved an inference speed of 88 FPS on a Tesla V100 GPU. Efficient inference, enabling rapid analysis of entire digital slides, which may contain hundreds of cells, without noticeable latency, is crucial for clinical applications. Potential workflow integration could enable CA-YOLO to serve as an automated pre-screening assistant. The system first scans the digital slide image and uses the CA-YOLO model to automatically detect and highlight all potential clue cells. These marked regions of interest are then presented to the expert, reducing manual search time and allowing them to focus on confirming diagnostically relevant cells, thereby improving the efficiency and reliability of bacterial vaginosis diagnosis.

## 8. Conclusions and Future Work

This paper proposes the CA-YOLO model, designed to address the significant challenge of automatically detecting clue cells in bacterial vaginosis diagnosis. By enhancing the YOLOv8 framework, we introduce three key innovations: the CAM captures the surface texture of bacterially adhered cells, the SGAM focuses on the morphology of ambiguous cell edges, and focal loss addresses the severe class imbalance. Experimental results demonstrate that CA-YOLO achieves a significant improvement in sensitivity, thereby providing a reliable tool for clinical screening by reducing missed diagnoses.

Despite its strong performance in sensitivity, we acknowledge key limitations. As discussed, the modest F1 score reflects a necessary trade-off between precision and recall, and the model’s robustness could be further enhanced by training on more diverse, multi-center datasets.

To address these points, our future work will proceed in two main directions. First, we will investigate advanced loss functions and post-processing techniques to improve the F1 score by achieving a better balance between precision and recall, without compromising sensitivity. Second, we will focus on expanding our dataset to enhance the model’s generalization capabilities. These efforts will be directed towards developing a more balanced, robust, and clinically deployable automated system for bacterial vaginosis diagnosis.

## Figures and Tables

**Table 1:** Hyper-parameter search space and selected best values for CA-YOLO training.

Hyper-Parameters	Search Space	Best Value
learning rate	[0.0001, 0.0005, 0.001, 0.005, 0.01]	0.001
batch size	[16, 32, 64]	64
weight decay	[0.0, 0.0005, $1\;\times \;10^{-5}$, $1\;\times \;10^{-4}$, $1\;\times \;10^{-3}$]	0.0005

**Table 2:** Performance comparison of placing the SGAM module at different locations.

Model Structures	SEN	SPE	ACC	F1
3_layers_P345	0.765 ± 0.020	0.929 ± 0.025	**0.903 ± 0.018**	0.442 ± 0.015
1_layer_P5	**0.778 ± 0.036**	**0.941 ± 0.016**	0.902 ± 0.016	**0.503 ± 0.025**

**Table 3:** Comparison of different attention mechanisms integrated into the CA-YOLO model.

Attention Modules	SEN	SPE	ACC	F1
SE [36]	0.762 ± 0.051	0.923 ± 0.015	0.894 ± 0.017	0.445 ± 0.023
CBAM [40]	0.728 ± 0.051	0.939 ± 0.005	0.901 ± 0.008	0.455 ± 0.019
ECA [49]	0.709 ± 0.071	0.930 ± 0.013	0.891 ± 0.005	0.458 ± 0.022
SimAM [50]	0.714 ± 0.049	0.926 ± 0.022	0.888 ± 0.013	0.444 ± 0.024
SGAM (proposed)	**0.778 ± 0.036**	**0.941 ± 0.016**	**0.902 ± 0.016**	**0.503 ± 0.025**

**Table 4:** Ablation study of the proposed CA-YOLO model components.

Model Structures	SEN	SPE	ACC	F1
w/o SGAM	0.766 ± 0.036	0.940 ± 0.014	**0.908 ± 0.011**	0.493 ± 0.011
w/o CAM	0.761 ± 0.031	0.936 ± 0.008	0.904 ± 0.009	0.488 ± 0.014
w/o focal loss	0.765 ± 0.045	0.939 ± 0.022	0.908 ± 0.014	0.480 ± 0.025
CA-YOLO	**0.778 ± 0.036**	**0.941 ± 0.016**	0.902 ± 0.016	**0.503 ± 0.025**

**Table 5:** Performance comparison with other object detection methods.

Models	SEN	SPE	ACC
YOLOv11-s [51]	0.686 ± 0.052	**0.963 ± 0.009**	0.904 ± 0.010
MSP-YOLO [25]	0.706 ± 0.029	0.961 ± 0.012	0.900 ± 0.006
CAF-YOLO [16]	0.531 ± 0.146	0.936 ± 0.013	0.860 ± 0.024
YOLO-TCT [17]	0.543 ± 0.037	0.936 ± 0.012	0.867 ± 0.010
CA-YOLO (v11)	0.765 ± 0.042	0.941 ± 0.005	**0.909 ± 0.003**
CA-YOLO	**0.778 ± 0.036**	0.941 ± 0.016	0.902 ± 0.016

**Table 6:** Statistical tests for comparing CA-YOLO performance with baseline models.

Statistical Test	Metric	Result	Conclusion
Bootstrap Test	95% CI for Accuracy Difference	[+1.75%, +7.43%]	Significant (Interval does not contain 0)
McNemar’s Test	*p*-value	*p* = 0.0035 (<0.01)	Significant (*p* < 0.05)

**Table 7:** Comparison of detection performance on the BCCD dataset.

Models	Precision	Recall	F1
YOLOv8-s [54]	0.537	0.898	0.672
BC-YOLO [53]	**0.901**	**0.941**	**0.920**
CA-YOLO	0.553	0.926	0.693

## Data Availability

Data will be made available upon reasonable request to the corresponding author.
